# Setting the standard for high-quality studies using open health datasets

**DOI:** 10.1371/journal.pmed.1004854

**Published:** 2025-12-04

**Authors:** Andreia Cunha, Suzanne de Bruijn, Alison Farrell, Helen Lumbard, Alexandra Tosun, Daniel Routledge

**Affiliations:** 1 Public Library of Science, Cambridge, United Kingdom; 2 Public Library of Science, San Francisco, United States of America; 3 Public Library of Science, Berlin, Germany

## Abstract

Large open health datasets present unique opportunities for studies that when well-designed, conducted, and reported, can offer valuable contributions to health and medicine. However, recent years have seen a concerning proliferation of analyses lacking robust or novel findings. This Editorial provides guidance to authors for conducting and reporting high quality secondary analyses using these datasets.

## Teaser

Large open health datasets present unique opportunities for studies that when well-designed, conducted, and reported, can offer valuable contributions to health and medicine. However, recent years have seen a concerning proliferation of analyses lacking robust or novel findings. In this Editorial, we provide guidance to authors for conducting and reporting high-quality secondary analyses using these datasets.

Much has been written about the value of open science and hypothesis-driven secondary analyses of open health datasets [1], and *PLOS Medicine* has been and continues to be a strong supporter of both. There is a growing ecosystem of these rich resources—including the UK Biobank [2], National Health and Nutrition Examination Survey [3], the Global Burden of Disease Study [4], and the Demographic and Health Surveys Program [5], to name a few. Open health datasets have the potential to both democratize science and tackle crucial health challenges. They provide valuable opportunities to the global scientific community to test hypotheses when generating and maintaining the data would otherwise not be feasible, such as in resource-constrained settings [6]. When carefully designed, analyses using such datasets have provided novel insights on important global and national health issues, for example, exploring the social determinants of health outcomes, assessing equity in access to care, or mapping disease burden across populations [7–10]. Well-conducted descriptive studies have demonstrated how socioeconomic status influences mortality, how health system performance varies across contexts, or how care cascades can reveal critical gaps in disease prevention and management [7,9,10].

While the value of data-driven health research using publicly available datasets is undeniable, a recent proliferation of poorly conducted analyses—including growing submissions from suspected paper mill operations [11,12]—has raised concerns that these studies threaten the integrity and value of scientific literature. Utilization of large datasets does not guarantee the quality of research; analyses are only as good as the underlying research questions, assumptions, biases, and representativeness of the data. Unfortunately, there is persistent variability in the methodological rigor of large open health data studies, with inconsistent application of robust statistical methods, suboptimal handling of missing data, and lack of transparency of data sources, analytical decisions, code, and study limitations. These issues have been exacerbated by the application of artificial intelligence to generate formulaic manuscripts reporting single associations without a strong scientific rationale, false-positive findings with inadequate adjustment for multiple testing, and/or selective use of subsets of data [11,12]. Although generating a paper quickly and easily using this approach can seem appealing in work cultures where publication numbers are rewarded, it is a damaging practice that clutters the scientific literature with contributions of little value, or misleading findings with potentially detrimental effects for clinical practice and public health.

To preserve the integrity and value of open health data research, clearer standards for publication are essential. In response to these challenges, several publishers, including PLOS, have announced new policies for retrospective studies using health databases [13,14]. PLOS journals will automatically reject such manuscripts unless researchers provide additional work, such as experiments or primary analyses that validate results and clearly establish their contribution to the field. In addition, the Journal of Global Health has developed guidelines aimed at mitigating the key negative impacts associated with such studies [6].

To assist authors in planning and conducting high-quality secondary analyses of open health datasets, and to provide clarity on editorial standards, we have prepared a 10-point guide outlining factors that contribute to a strong study (see Box 1).

**Box 1. Considerations for generating high-quality secondary analyses of open health datasets**

1. QUESTION: Formulate a broad research question of current relevance to clinical practice or policy, strongly grounded in biological or social theory, and *then* choose the most suitable datasets to answer it.
2. IMPACT: Articulate the specific ways in which the study drives progress on a significant medical or public health problem, including its potential to influence clinical practice, service delivery, or policy decision-making.
3. NOVELTY: Explain what is genuinely new about the work—whether in the question, data, method, or insight—and how it offers value that cannot be obtained from existing publications, datasets, or readily accessible tools.
4. DATA: Understand the constraints of the datasets, such as their age, number of time-points, missing data, relevance of health codes, categorization of phenotypes, global representation, and how these limitations will affect the analysis.
5. PRE-REGISTRATION: Plan the analysis and pre-register the study protocol, which will help prevent data analyses without a relevant, pre-specified question, as well as reduce duplication of work by independent researchers.
6. COLLABORATION: Enlist collaborators that complement the authors’ expertise either in methodology, practice, or policy to maximize the potential that the data analysis will produce actionable insights.
7. COMPREHENSIVENESS: Perform analyses that are comprehensive across a given dimension, for example, all relevant exposures or outcomes, which will reduce the risk of selective reporting.
8. METHODOLOGY: Have a planned strategy to mitigate confounding, including choosing the most appropriate statistical analyses, using populations with different confounding structures, and/or performing sensitivity analyses.
9. VALIDATION: Use multiple datasets to replicate the findings and demonstrate broader relevance and generalizability, as well as ensure statistical power.
10. REPORTING: Ensure adherence to the highest standards of methodological rigor and community-endorsed checklists in any study design, particularly when performing observational analyses with claims of potential causality.

These recommendations are not intended to be overly prescriptive but rather act as guidance for authors whilst planning and conducting their study. At *PLOS Medicine*, we will maintain a high editorial bar for the quality of submissions using open health data, facilitate code sharing and publication of community-endorsed reporting checklists, but most importantly, we will adapt as tools and datasets evolve, ensuring a robust standard of methodological review and scientific integrity. We maintain that studies reporting secondary analyses of open health datasets, when well-designed, conducted, and reported, can offer a valuable contribution to our understanding of human health and disease, and inform clinical and public health practice and policy. We therefore remain committed to supporting authors and welcoming submissions of novel, high-quality, rigorous analyses of publicly available health datasets.
